# Inflammation Drives Dysbiosis and Bacterial Invasion in Murine Models of Ileal Crohn’s Disease

**DOI:** 10.1371/journal.pone.0041594

**Published:** 2012-07-25

**Authors:** Melanie Craven, Charlotte E. Egan, Scot E. Dowd, Sean P. McDonough, Belgin Dogan, Eric Y. Denkers, Dwight Bowman, Ellen J. Scherl, Kenneth W. Simpson

**Affiliations:** 1 Department of Clinical Sciences, College of Veterinary Medicine, Cornell University, Ithaca, New York, United States of America; 2 Department of Microbiology and Immunology, College of Veterinary Medicine, Cornell University, Ithaca, New York, United States of America; 3 MR DNA (Molecular Research), Shallowater, Texas, United States of America; 4 Department of Pathology, College of Veterinary Medicine, Cornell University, Ithaca, New York, United States of America; 5 Division of Gastroenterology and Hepatology, Jill Roberts Inflammatory Bowel Disease Center, Weill Cornell Medical College, Cornell University, New York, New York, United States of America; Charité, Campus Benjamin Franklin, Germany

## Abstract

**Background and Aims:**

Understanding the interplay between genetic susceptibility, the microbiome, the environment and the immune system in Crohn’s Disease (CD) is essential for developing optimal therapeutic strategies. We sought to examine the dynamics of the relationship between inflammation, the ileal microbiome, and host genetics in murine models of ileitis.

**Methods:**

We induced ileal inflammation of graded severity in C57BL6 mice by gavage with *Toxoplasma gondii*, *Giardia muris*, low dose indomethacin (LDI;0.1 mg/mouse), or high dose indomethacin (HDI;1 mg/mouse). The composition and spatial distribution of the mucosal microbiome was evaluated by 16S rDNA pyrosequencing and fluorescence in situ hybridization. Mucosal *E. coli* were enumerated by quantitative PCR, and characterized by phylogroup, genotype and pathotype.

**Results:**

Moderate to severe ileitis induced by *T. gondii* (day 8) and HDI caused a consistent shift from >95% Gram + *Firmicutes* to >95% Gram - *Proteobacteria*. This was accompanied by reduced microbial diversity and mucosal invasion by adherent and invasive *E. coli*, mirroring the dysbiosis of ileal CD. In contrast, dysbiosis and bacterial invasion did not develop in mice with mild ileitis induced by *Giardia muris*. Superimposition of genetic susceptibility and *T. Gondii* infection revealed greatest dysbiosis and bacterial invasion in the CD-susceptible genotype, NOD2^−/−^, and reduced dysbiosis in ileitis-resistant CCR2^−/−^ mice. Abrogating inflammation with the CD therapeutic anti-TNF-α-mAb tempered dysbiosis and bacterial invasion.

**Conclusions:**

Acute ileitis induces dysbiosis and proliferation of mucosally invasive *E. coli,* irrespective of trigger and genotype. The identification of CCR2 as a target for therapeutic intervention, and discovery that host genotype and therapeutic blockade of inflammation impact the threshold and extent of ileal dysbiosis are of high relevance to developing effective therapies for CD.

## Introduction

Inflammatory bowel diseases (IBD) encompass a complex of inflammatory intestinal disorders that are increasing in global prevalence and typified by recurrent flares of acute on chronic inflammation. [1], [2] Traditionally, IBD comprises two distinct entities, Crohn’s Disease (CD) and ulcerative colitis (UC), [1], [2], [3] both widely accepted to arise from the convergence of genetic susceptibility, enteric bacteria, the immune response, and environmental factors such as smoking, stress and diet. [3]–[10].

The best-characterized genetic susceptibility locus in CD has been mapped to NOD2, with polymorphisms reported in 20–30% of CD patients. [11] The NOD2 gene encodes an intracellular pathogen recognition receptor that, on sensing the bacterial muramyl dipeptide, evokes a pro-inflammatory response mediated by NF-B. [12], [13] The effect of NOD2 polymorphisms in CD are incompletely understood, but appear to culminate in impaired bactericidal defenses and unchecked intestinal inflammation. [14], [15] More recently, genome-wide studies have shown CD-associated polymorphisms in autophagy-related 16-like protein 1 (ATG16L1) and immunity-related GTPase family, M, (IRGM), genes involved in killing intracellular microbes. [6], [16], [17] Clearly, the emerging theme in CD genetics involves the abnormal interfacing of innate immunity with intestinal microbes.

Bacteria play a pivotal role in CD pathogenesis, purportedly via loss of immune tolerance to endogenous flora. [2], [4], [18], [19] However more recent work shows global imbalances of the intestinal microbiome in CD, termed ‘dysbiosis,’ typified by a predominance of ‘aggressive’ species such as *Proteobacteria* relative to ‘protective’ species such as *Firmicutes.* A novel pathotype within *Proteobacteria,* Adherent and Invasive *E. coli* (AIEC), is increasingly associated with CD, and can invade and persist intracellularly within epithelial cells and macrophages. [20]–[23] A role for AIEC in CD is further supported by their ability to induce granulomas *in vitro* and to exploit host defects conferred by CD-associated polymorphisms in ATG16L, IRGM and NOD2. [24], [25].

Here we examine the dynamics of the relationship between inflammation and the ileal microbiome in murine models of ileitis incorporating environmental triggers and pathomechanisms of relevance to CD: *Toxoplasma gondii*, *Giardia muris* and indomethacin. While *T. gondii* is not typically associated with CD, it induces granulomatous ileitis of Th1-type immunopathology in C57BL/6 mice that mimics ileal CD. [26], [27] The development of inflammation in this model is also microbial dependent. [27], [28] Giardia muris infection is associated with mild to moderate small intestinal inflmmation characterized by intraepithelial cell infiltration, altered villus morphology, decreased disaccharidae activity and T cell dependence. [29], [30] Indomethacin induces dose-dependant small intestinal damage in mice that involves the enteric flora, cytokines such as TNF-a, and TLR-4 mediated signaling. [31].

We show that moderate to severe ileitis is induced by *T. gondii* and indomethacin, and causes a consistent pattern of dysbiosis, characterized by reduced microbial diversity and a global shift in the ileal microbiome from >95% Gram +, to >95% Gram - species. Mucosal invasion by *E. coli* with an AIEC-pathotype accompanies severe ileitis and dysbiosis. In contrast, dysbiosis and bacterial invasion did not develop in mice with mild ileitis induced by *Giardia muris*. Next we show that dysbiosis induced by *T. gondii* is significantly muted and bacterial invasion prevented when we limit the inflammatory response via deletion of pro-inflammatory chemokine receptor, CCR2. In contrast, we observe heightened dysbiosis and *E. coli* invasion when we induce ileitis in the absence of NOD2. Lastly, abrogating inflammation with anti-TNF-α, a mainstay of CD management, limits the extent of dysbiosis and bacterial invasion. In summary, we establish that acute ileitis induces dysbiosis and proliferation of mucosally invasive *E. coli,* irrespective of trigger and genotype. We discover that therapeutic blockade of inflammation may indirectly control dysbiosis, and speculate that failure to completely resolve acute dysbiosis may set the scene for chronic microbial-driven inflammation in CD.

## Materials and Methods

### Mice

Eight to 12 week old female C57BL/6 and Swiss Webster mice were purchased from Jackson Laboratory and Taconic Farms. Breeding pairs of CCR2^−/−^ and Nod2^−/−^ mice were purchased from The Jackson Laboratory. Mice were established under specific pathogen-free conditions in the Transgenic Mouse Facility at Cornell University, which is American Association of Laboratory Animal Care accredited. Samples of ileum were obtained after rinsing the intesine of fecal contents with 10 ml sterile PBS introduced via a sterile gavage needle.

### Toxoplasma Gondii Infections

Type II *T. gondii* cysts (ME49) were obtained from chronically infected Swiss Webster mice by homogenizing brains in sterile PBS. Groups of age-matched C57BL/6 wild type, CCR2^−/−^ and Nod2^−/−^ mice were infected with 100 cysts by gavage, and ileum harvested at 4 (n = 5/group, T4) and 8 (n = 5/group, T8) days post-infection. Five uninfected control mice were gavaged with sterile PBS and ileum harvested on day 8. Samples of ileum were obtained after rinsing the intestines of fecal contents with 10 ml sterile PBS introduced via a sterile gavage needle.

### Indomethacin

Indomethacin solubilized in PBS was administered by gavage to C57BL/6 mice at a low dosage (LDI) of 0.1 mg/mouse/day (n = 5) for 5 days, and a high dosage (HDI) of 1 mg/mouse/day for 3 days (n = 5). Ileum was harvested on day 7 (LDI) and day 3 (HDI).

### Giardia Muris Infections

2×10^5^ *G. muris* trophozoites were administered to C57BL/6 mice by gavage. Ileum was harvested at 7 (n = 5, G7) and 14 days (n = 5, G14) post-infection. Five uninfected control mice received sterile PBS by gavage and ileum was harvested on day 14.

### Anti-TNF-α mAb Treatment

Rat anti-mouse TNF-α (clone XT22.11) was purified from hybridoma supernatants by passage over a protein G sepharose column (Invitrogen). Mice were injected intraperitoneally with 3 mg anti-TNF-α or 3 mg of control rat IgG (Jackson ImmunoResearch) on days 3, 5 and 7 post- *T. gondii* infection of mice from Taconic Farms.

### Histopathology

Hematoxylin and eosin stained ileal sections were examined by a blinded pathologist. An ileitis score (0 to 9) was calculated for each section by summing the degree of cellular infiltration (0 = normal, 1 = minimal, 2 = moderate, 3 = severe), extent of architectural abnormality (0 = normal, 1 = minimal, 2 = moderate, 3 = severe), and the presence or absence of ulcers, necrosis and neutrophils (0 or 1). Villus height and crypt depth were measured and a crypt:villus ratio calculated for each section.

### Pyrosequencing

Pyrosequencing was performed using the bTEFAP method. [32], [33], [34] DNA was amplified using 27F-519R primers, labeled with linkers and tags, and pyrosequencing performed by Titanium chemistry FLX sequencing (Roche Applied Science, Indianapolis, IN). Data were curated and only high quality sequence reads (Phred20) used, with the final data annotated with BLASTn and analyzed as described previously. [32], [33], [34].

### Fluorescence in situ Hybridization (FISH)

Formalin-fixed, paraffin-embedded sections of ileum mounted on Probe-On Plus slides (Fisher Scientific, Pittsburgh, PA, USA) were screened by FISH with probes targeting 16S rDNA for eubacteria, *E. coli* and Enterobacteriaceae (IDT, Coralville, IA, USA) as previously described. [20], [35] Sections were examined with an Olympus BX51 epifluorescence microscope. Images were captured with a DP-70 camera and processed using DP Manager (Olympus America, Center Valley, PA, USA).

### Quantitative PCR

Total bacteria and *E. coli* (uidA gene) were quantified as previously described. [20], [35], [36] A standard curve was generated using DNA from *E. coli* of known concentration. Bacterial DNA was normalized to biopsy size using murine 18S rRNA (Eurogentec, Seraing, Belgium). Bacterial number was expressed as colony-forming unit (CFU)/10^6^ murine cells.

### E. coli Characterization

*E. coli* strains were cultured from PBS-rinsed ileum as previously described. [35] Three to 5 *E. coli* colonies per biopsy were screened by RAPD-PCR and the major phylogenetic groups determined by triplex PCR. [20] Isolates differing in overall genotype were selected for subsequent analyses and stored at −80°C. Fresh non-passaged bacteria were used throughout. Isolates were serotyped and screened for heat-labile toxin, heat-stable toxins a and b, Shiga-like toxin types 1 and 2, cytotoxic necrotizing factors 1 and 2, and intimin-*γ* at the *E. coli* Reference Center, Penn State University. [37].

The presence of additional genes related to virulence in *E. coli* was determined by PCR (Table S6). [20], [38], [39], [40].

The ability of *E. coli* isolates to invade Caco-2 cells, and replicate in J774 macrophages was determined as previously described. [20], [35].

### Statistical Analysis

For comparisons involving >2 groups, Kruskal-Wallis and Mann-Whitney post-test were applied (non-parametric data) or ANOVA with Tukey Kramer post hoc analysis (parametric data). Comparisons between 2 groups were performed using Mann-Whitney. Pyrosequencing data was analyzed as described in Supplementary Methods available online. Chi-square was used to create the association plots for microbial families as described in Supplementary Methods available online. For all evaluations, p<0.05 was considered significant.

## Results

### T. gondii and Indomethacin Trigger Acute Ileitis

**Figure 1:**
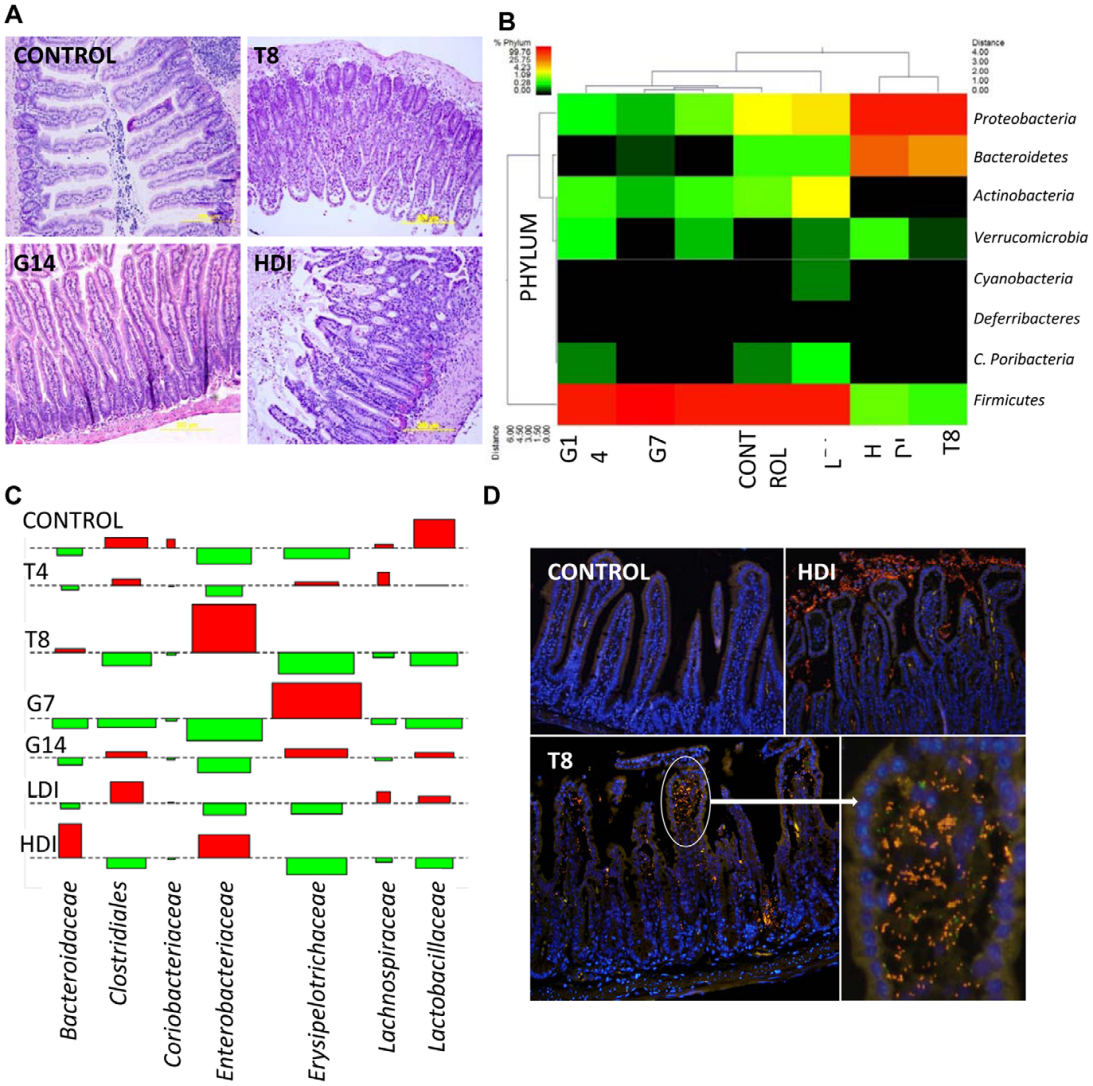
*T. gondii* infection and high dose indomethacin trigger severe ileitis, dysbiosis and mucosal *E. coli* invasion in C57BL/6 mice. (a) Histopathology. Normal ileal histology (inflammation score median  = 0, range 0–0) in control mice. Moderate to severe ileitis (4, 3–6) develops in *T. gondii* mice at 8 days post-infection (T8). *G. muris*-infected mice develop minimal inflammation (1, 1–1) and mucosal hyperplasia 14 days (G14). High dose indomethacin (HDI) induces severe ileitis (5, 5–7) (H&E 40x). (b) Pyrosequencing reveals a Gram− shift associated with ileitis. Gram+ bacteria predominate in control mice and mice with mild inflammation (G7, G14: Giardia 7 and 14 days p.i., T4: *T. gondii* 4 days p.i.; LDI: low-dose indomethacin). A shift to >95% Gram− bacteria dominated by the phyla *Proteobacteria* and *Bacteroidetes* (p<0.0001) is associated with moderate to severe ileitis in T8 and HDI mice. (c) Association plots of the number of sequences obtained by pyrosequencing corresponding to bacterial families. The area of each rectangle is proportional to the difference in observed and expected frequencies on chi square analysis. The observed frequency of a rectangle is indicated by position relative to baseline, increased (shaded red) or decreased (shaded green) highlighting the ileitis-associated shifts from *Firmicutes (Lactobacillaceae, Clostridiales)* to *Enterobacteriaceae* (T8, HDI) and *Erysipelotrichaceae* (G7, G14, T4). (d) Eubacterial FISH reveals scant luminal and mucosa-associated flora in control mice (EUB338-Cy3, non-EUB338-6FAM with DAPI counterstaining, 40x). Ileitis in T8 and HDI is associated with increased mucosal bacteria and invasive *E. coli* (*E*. *coli*-Cy3, EUB338-6FAM, 40x).

Granulomatous ileitis is present in approximately 70% of CD patients, thus our first aim was to establish clinically relevant murine models of ileitis using enteric infections (*T. gondii*, *G. muris*) and NSAID ingestion (indomethacin). *T. gondii* is known to induce ileitis with CD-like immunopathology in C57BL/6 mice, i.e. CD4^+^ T-cell mediated inflammation dominated by Th1 cytokines TNF-α and IFN-γ. [41], [42] We show that peroral infection with *T. gondii* strain ME49 in C57BL/6 mice induces moderate to severe ileitis (ileitis score 4,range 3–6) within 8 days of infection (T8, Figure 1a). Histologic changes (summarized in Table S1) comprised granulomatous inflammation, villus atrophy, crypt hyperplasia, and Paneth cell and goblet cell depletion, comparable to CD. [2], [42] In contrast, *G. muris* induced minimal to mild ileitis (ileitis score 1, range 1–1) and mucosal hyperplasia within 14 days of infection (G14, Figure 1a). Peroral indomethacin induced dose-dependent injury; low dosage (LDI) caused mild to moderate ileitis (ileitis score 2, range 1–4), whereas high dosage (HDI, Figure 1a) caused death in 2 mice, and ileal ulceration and necrosis of crypt cells in one and three of the survivors respectively (ileitis score 5, range 5–7). These results establish that we can induce ileitis of graded severity using environmental triggers of relevance to CD.

### Acute Ileitis is Associated with Dysbiosis and *E. coli* Invasion

To capture the microbial response to induced inflammation, we analyzed the composition and spatial distribution of the ileal microbiome by pyrosequencing and FISH analysis. The ileum of healthy C57BL/6mice, was dominated by Gram+ bacteria, with 98% of sequences classed as phylum *Firmicutes,* (Figure 1b, Tables S2 and S3). Moderate to severe ileitis in the *T. gondii* (T8) and indomethacin (HDI) models induced a dramatic shift (p<0.0001) to >99% Gram- bacteria, as depicted in the pyrosequencing dend gram (Figure 1b) and the association plot (Figure 1c). In T8, 92% of sequences were classed as *Proteobacteria* and 7% *Bacteroides.* In HDI, 75% of sequences were classed as *Proteobacteria* and 24% *Bacteroidetes.* Ileitis was also associated with marked loss of microbial diversity, from 25 genera in controls (97% of maximum predicted genera), to 11 and 13 genera in T8 and HDI (100% of maximum predicted genera) respectively (Table S5 and Figure S1). These observations were confirmed and extended by FISH analysis, which revealed large numbers of mucosa-associated bacteria and invasive *E. coli* in all T8 and HDI mice (Figure 1d).

In contrast, dysbiosis and bacterial invasion did not develop in mice with minimal (G7, G14) or mild (T4, LDI) ileitis (Figure 1d). We conclude that moderate to severe ileitis induces dysbiosis and *E. coli* invasion in the absence of genetic susceptibility.

### Severe Ileitis is Associated with E. coli Proliferation

**Figure 2:**
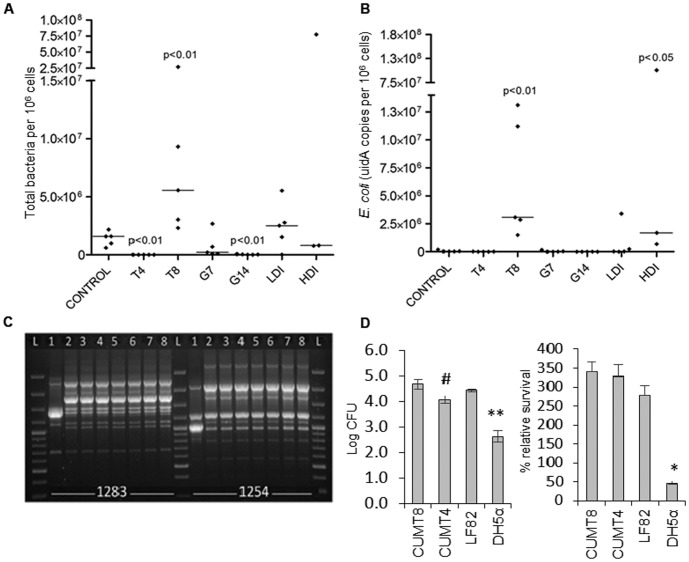
Ileitis-induced dysbiosis is associated with increased bacteria and clonal proliferation of adherent invasive *E. coli* (AIEC). (a) Total bacteria quantified by real-time PCR, expressed as bacterial CFU per 10^6^ murine cells. Moderate to severe ileitis in T8 is associated with increased bacteria relative to controls. Mild pathology (T4, G14) induces a decrease. (b) Moderate to severe ileitis (T8, HDI) induces *E. coli* proliferation (*E. coli uidA* quantification by real-time PCR). (c) Agarose gel electrophoresis showing the products of Random Amplification of Polymorphic DNA (RAPD-PCR) using primers 1283 and 1254. *E. coli* strains from: T4 (Lanes 1–3), T8 (4, 5) and HDI (6–8). Lane L, 100bp plus DNA ladder. *E. coli* isolates in lanes 2–8 are clonal, and a representative strain was designated CUMT8. *E. coli* in lane 1 was designated CUMT4. (d). Invasion and survival of *E. coli* CUMT8 and CUMT4 in cultured epithelial cells (Caco-2) and macrophages (J774). CUMT8 and CUMT4 invade, and persist intracellularly like CD-associated AIEC LF82, and better than commensal *E. coli* DH5α (* = P<0.01, ** = P<0.001 vs other strains). CUMT4 was less invasive than CUMT8 (# =  P<0.01).

We next quantified the numbers of total bacteria and *E. coli* in ileal tissue by real-time PCR. [20], [36] In T8, the total bacterial load increased relative to controls (p<0.01), as did *E. coli* (p<0.01, Figure 2a, b). In addition, total bacterial load and *E. coli* were significantly greater in T8 versus T4 (total bacteria, T8 vs T4 p<0.01, and *E. coli*, T8 vs T4 p<0.01). In HDI there was a significant increase in *E. coli* (p<0.05). In contrast, in mice with minimal to mild ileal pathology (G14, T4), the median total bacterial counts were lower than controls (p<0.01) and *E. coli* did not increase. In summary, inflammation modulates the number of total bacteria and *E. coli*.

### Ileitis-associated E. coli Strains are Clonal and Display an Adherent and Invasive Pathotype

*E. coli* was cultured from 2/5 T4 mice with mild ileitis, and 8/8 mice (T8 and HDI) with moderate to severe ileitis. All 32 *E. coli* isolates were phylogroup B1, and 29 had an identical RAPD banding pattern (Figure 2c). Representative strains from the dominant and minor RAPD groups were designated CUMT8 (serotype 08:H21) and CUMT4 (serotype 055:H8) respectively. CUMT4 was isolated from a single mouse (T4) co-colonized with CUMT8 (Figure 2b). Both CUMT8 and CUMT4 lacked the common virulence genes found in pathogenic *E. coli*, and carried genes associated with AIEC (hemolysin co-regulated protein, long polar fimbriae, and a Type II secretion system). [20], [35] Phenotypically, CUMT8 and CUMT4 behaved like the CD-associated AIEC strain LF82, and were able to invade epithelial cells and persist within macrophages (Figure 2d). [21] These results demonstrate that ileitis of different etiologies induces proliferation of Adherent and Invasive *E. coli*.

### Abrogating Ileitis by CCR2 Deletion Limits Dysbiosis and Prevents E. coli Invasion

**Figure 3:**
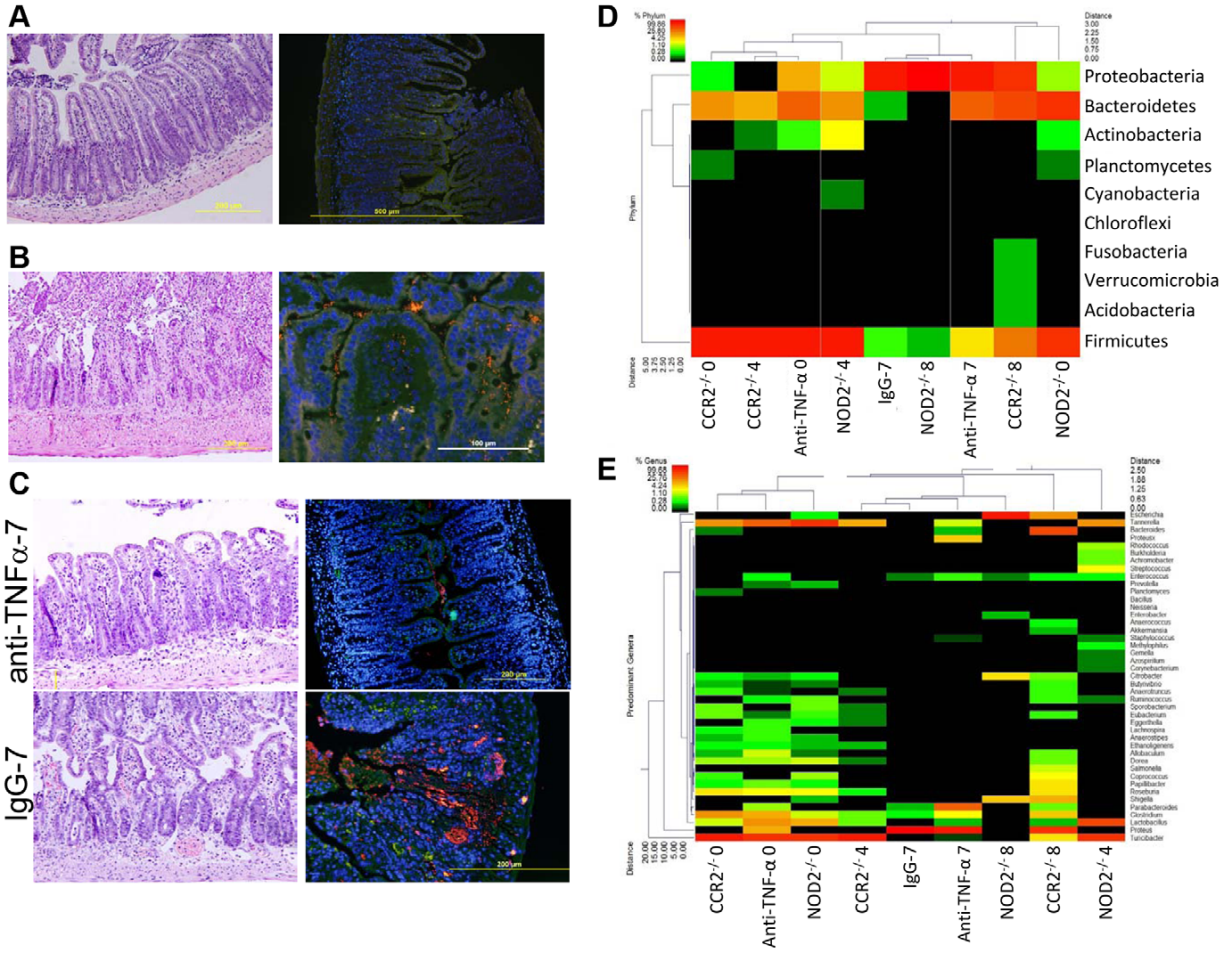
Dysbiosis and *E. coli* invasion are modulated by genetic susceptibility and pharmacotherapy. (a) CCR2^−/−^8 (n = 5) are protected from *T. gondii-*induced ileitis (median inflammation score 3, range 2–3) and *E. coli* invasion. (b) NOD2^−/−^8 (n = 5) develop severe ileitis (5, 5–7) and *E. coli* invasion. (c) anti-TNF-α mAb-7 (n = 5) reduces ileitis (4, 2–4 vs 4, 4–7) and decreases bacterial invasion relative to IgG-7 controls, n = 5). Histology, H&E 40x; FISH, CCR2^−/−^8 and anti-TNF-α-7 =  Cy3-EUB338/non-EUB338-6FAM; NOD2^−/−^8 and IgG-7 =  Cy3-*E. coli*/EUB338-6FAM, 40x. (d,e). Pyrosequencing reveals that *T. gondii*-induced dysbiosis is modulated by genetic susceptibility and pharmacotherapy. Abrogating ileitis maintains microbial diversity (CCR2^−/−^8), whereas enhancing ileitis decreases diversity and increases *E. coli* (NOD2^−/−^8). NOD2 deletion is associated with a baseline shift to *Bacteroidetes* (NOD2^−/−^0). Anti-TNF-α mAb temper dysbiosis (anti-TNF-α-7 versus IgG-7).

To further explore the interdependence of dysbiosis and inflammation, we evaluated the ileal microbiome in the setting of a limited host immune response, utilizing the *T. gondii* trigger model and CCR2^−/−^ mice. Polymorphisms in CCR2 are associated with CD, and mice lacking the pro-inflammatory chemokine receptor CCR2 are protected from *T. gondii*-induced intestinal damage. [41] Here, ileitis in CCR2^−/−^ at day 8 post-infection (CCR2^−/−^8) was less severe (P<0.05) than T8 (Figure 3a, Table S1). Pyrosequencing in uninfected CCR2^−/−^ (CCR2^−/−^0) revealed 91% *Firmicutes,* and 8% *Bacteroidetes* (Figure 3d, Tables S3 and S4). In CCR2^−/−^8, 58% of sequences were G- *Proteobacteria*, 27% *Bacteroidetes* and 14% *Firmicutes*. Despite this marked shift from 95% Gram+ flora in CCR2^−/−^0, to 84% Gram- flora in CCR2^−/−^8 (p<0.0001), microbial diversity was actually increased in CCR2^−/−^8, with 22 genera present (89% of maximum predicted genera) at day 8 versus 18 genera (90% of maximum predicted genera) at day 0 (Figure 3e). FISH revealed minimal mucosa-associated bacteria and no bacterial invasion in CCR2^−/−^0 and CCR2^−/−^8 (Figure 3a). Real-time PCR showed significantly (p<0.05) increased *E. coli* at day 8 (median: day 8 = 9.16×10^4^, vs Day 0 = 8.8×10^3^) but importantly this was much less (p<0.01) than in T8 (median 3.1×10^6^) and HDI (1.68×10^6^, Figure 2b). A single *E. coli* strain isolated from one CCR2^−/−^8 mouse was clonal with CUMT8. We have shown that abrogating inflammation limits dysbiosis by maintaining microbial diversity, controlling bacterial proliferation, and preventing *E. coli* invasion.

### The CD-susceptible Genotype NOD2^−/−^ Lowers the Threshold for Dysbiosis

To evaluate the impact of a CD-susceptible genotype on the inflammatory microbiome, we applied the *T. gondii* ileitis model to NOD2^−/−^ mice. This resulted in development of ileitis at day 4 (ileitis score 2, range 1–4) and severe histiocytic and neutrophilic inflammation (ileitis score 5, range 5 to 7) by day 8 (NOD2^−/−^8, Figure 3b). Pyrosequencing of uninfected mice showed a shift in the endogenous flora, with 53% *Firmicutes* and 46% *Bacteroidetes* in NOD2^−/−^0, relative to 98% *Firmicutes* in uninfected wildtype mice (Figure 3d, 3e, Tables S3 and S4). By day 8 post-infection we observed a dramatic shift to 99.8% *Proteobacteria*. The diversity of the primary populations also decreased markedly, from 19 in NOD2^−/−^0 (88% of maximum predicted genera), to only 6 genera in NOD2^−/−^8 (100% of maximum predicted) as shown in Figure 3e. Large numbers of mucosa-associated bacteria and invasive *E. coli* were observed in all NOD2^−/−^8 mice (Figure 3b), and *E. coli* increased from 1.13×10^3^ uidA copies/10^6^ cells in NOD2^−/−^0, to 1.19×10^4^ in NOD2^−/−^8 (P<0.05). These results reveal that NOD2^−/−^ perturbs the endogenous flora and enhances inflammation and dysbiosis in response to an injurious trigger.

### Anti-TNF-α mAb Limits Dysbiosis and Reduces Bacterial Invasion

Biological agents targeting the pro-inflammatory cytokine tumor necrosis factor alpha (TNF-α), are increasingly used in the management of CD. [42] Thus, our final aim was to explore the impact of anti-TNF-α mAb on the inflammation-dysbiosis dynamic. Mice treated with anti-TNF-α mAb developed less severe inflammation 7 days after *T. gondii* infection (ileitis score 4, range2–4)) than controls (ileitis score 4, range 4–7) receiving an irrelevant antibody (IgG-7, Figure 3c).

Pyrosequencing revealed a marked floral shift and loss of microbial diversity, from 71% *Firmicutes*, 21% *Bacteroidetes*, 6% *Proteobacteria* and 21 genera in uninfected controls (anti-TNF-α-0), to 99.7% *Proteobacteria* (99% *Proteus* spp.*),* and 4 genera in the IgG-7 group (Figure 3d, 3e, Tables S3 and S4). In anti-TNF-α-7, dysbiosis and loss of microbial diversity were tempered: 72% *Proteobacteria* (72% *Proteus spp.*), 21% *Bacteroidetes*, and 10 genera. We observed increased mucosa-associated and invasive bacteria in IgG-7 (5/5 mice with bacterial invasion) relative to anti-TNF-α-0 (0/5 mice), and anti-TNF-α-7 (low numbers of intramucosal bacteria in 3/5 mice) (Figure 3c). Interestingly, the vast majority of invasive bacteria in IgG-7 and anti-TNF-α-7 didn’t hybridize with an *E. coli* probe. Based on pyrosequencing and bacterial culture, we suspect these invasive bacteria are *Proteus* spp. This contrasts with invasive *E. coli* in T8 and HDI, and likely reflects the relative abundance of *Proteus* in the endogenous flora of mice in this experiment that were purchased from Taconic Farms vs. Jackson Laboratories. These results show that anti-TNF-α treatment while not completely protective, decreases the inflammation-induced floral shift by maintaining greater microbial evenness and reducing bacterial invasion.

## Discussion

The evidence is compelling that CD pathogenesis involves interplay between the intestinal microbiome, genetic susceptibility, the immune system, and environmental risk factors. Our limited understanding of these interrelationships has yet to reconcile the wide spectrum of CD phenotypes, but mapping their interactions is pivotal to understanding CD pathogenesis and identifying new therapeutic targets. Here we establish that acute ileitis induces a consistent shift in the microbiome from *Firmicutes* to *Proteobacteria*, accompanied by a reduction in microbial diversity and proliferation of AIEC. This ‘inflammatory microbiome’ recapitulates the dysbiosis of ileal CD. [20], [43], [44], [45] When we superimposed a CD-susceptible genotype, NOD2^−/−^, on *T. gondii*-induced ileitis, we enhanced inflammation and dysbiosis. Conversely, dysbiosis was tempered when we limited inflammation by CCR2 gene deletion, revealing a new potential therapeutic target in CD. Using anti-TNF-α mAb, a mainstay of CD therapy, we discover that dysbiosis can be reduced by pharmacologic manipulation of mucosal inflammation. We speculate that failure to resolve the inflammatory microbiome after acute non-specific enteric injury may stimulate persistent microbial-drive inflammation in CD.

**Figure 4:**
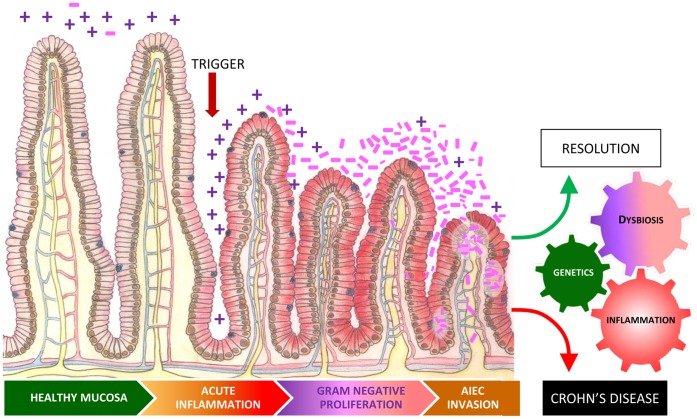
Inflammation drives dysbiosis, Gram negative proliferation and *E. coli* invasion. Independent of genotype, ileitis induces a progressive decrease in microbial diversity, a shift from *Firmicutes* (Gram**+**) to *Proteobacteria* (Gram−), and proliferation of AIEC. Superimposition of genetic susceptibility can lower (NOD2^−/−^) or increase (CCR2^−/−^) the threshold for dysbiosis in response to an external trigger. We speculate that genetic susceptibility may also influence the ability of an individual to resolve the self-perpetuating cycle of dysbiosis and inflammation generated by an acute insult.

Many IBD susceptibility genes have been discovered, but similar advances in defining environmental risk factors have lagged. [8] Animal models of CD are usually chemically-induced, genetically engineered, or congenic rodent strains with limited relevance to the clinical setting and disease phenotype. [3], [46] To specifically examine the interrelationship between the microbiome and inflammation in ileal CD, we utilized murine models of ileitis incorporating environmental triggers of relevance to CD. NSAID ingestion, infectious enteritis and stress are known IBD risks. [47], [48] [10] While *T. gondii* is not typically associated with CD, it induces granulomatous ileitis of Th1-type immunopathology in C57BL/6 mice that mimics ileal CD. [26], [27] The development of inflammation in this model is also microbial dependent. [27], [28] Using these diverse stimuli we showed that the inflammatory microbiome is a common endpoint of ileitis, driven by severity of inflammation rather than the trigger. Our finding that mild ileitis (T4, G14) was associated with decreased bacterial load suggests an ability to limit dysbiosis until inflammation overwhelms. The mucosal hyperplasia in G14 mice raises the possibility that up-regulation of anti-microbial mucosal defenses may occur with mild injury. [49].

The question that naturally arises is whether the floral shift *per se* is in fact harmful, or a benign consequence of inflammation. However, consider that we show the degree of dysbiosis to directly relate to the severity of ileitis, and that invasive *E. coli* is always, and only, present in mice that develop a >95% G- shift. Further, the selection pressure exerted by inflammation is sufficiently strong to drive clonal proliferation of a single AIEC strain (CUMT8) in all mice with severe ileitis, independent of stimulus. Here we show for the first time that acute ileitis is closely linked to the proliferation of mucosally invasive *E. coli* with an AIEC pathotype. [20], [21], [35].

Increasingly implicated in CD in the US and Europe, AIEC have been isolated from the ileum of 36–38% of CD patients versus 6% of controls, and are associated with the severity of ileal CD. [20], [50] Surprisingly little is known about the luminal microenvironment that promotes the proliferation of *E. coli* and disappearance of *Firmicutes* such as *Fecalibacterium prauzsnitzii*. A plethora of inflammation-associated factors could favor, or fail to regulate bacterial proliferation and virulence. [20], [23], [24], [50] Specific mechanisms implicated in the adhesion and invasion of AIEC include bacterial factors such as flagellin [23] and Type I pili,[51] as well as host factors such as cell adhesion molecule CEACAM6[52] and the stress response protein Gp96. [53] Up-regulation of CEACAM6 and interaction with type 1 pili to promote adhesion and invasion is a potential pathomechanism for AIEC, but cannot account for our findings since mice do not express CEACAM6. [52] Notably, the highest *E. coli* counts were observed in mice with the most severe inflammation and dysbiosis, suggesting that quantification of mucosal *E. coli* may be a useful adjunct for monitoring CD activity. [20].

When we superimposed the effect of genotype on the inflammation-dysbiosis dynamic, we saw the most severe ileitis, dysbiosis, and *E. coli* invasion, in NOD2^−/−^ mice. We postulate that this is attributable to altered sensing and reduced killing of luminal and intracellular *E. coli*. [11] Our observation of more *Bacteroidetes* sequences in uninfected NOD2^−/−^ than other control groups suggests a pre-existing baseline floral shift. [54] Our findings indicate that NOD2 mutation confers greater vulnerability to inflammation and dysbiosis but importantly, absence of NOD2 was not a prerequisite for dysbiosis and invasion. This echoes the scenario in CD, where 60%–70% of CD patients show no NOD2 mutations. [11], [55] Moreover, studies of identical twins elegantly show that microbial composition is determined by presence or absence of CD rather than genetic make-up alone. [45], [56].

The interdependence of inflammation and dysbiosis is further supported by our findings in inflammation resistant CCR2^−/−^ mice. CCR2 is a chemokine receptor for monocyte chemoattractant proteins (MCP1–4) and we have shown previously that mice lacking CCR2 are protected from *T. gondii*-induced ileal pathology. [41] The CCR2^−/−^ model is relevant to CD pathogenesis because CCR2+ lamina propria lymphocytes are a specific feature of ileal CD [57] and polymorphisms in CCR2 have been associated with CD in several studies. [58], [59] On this basis we suggest that pharmacologic blockade of this receptor, which has been demonstrated in mice, [60] may provide a novel pathway for therapeutic intervention in CD. In a similar vein, by controlling inflammation using anti-TNF-α mAb, we reduced dysbiosis, unmasking an effect of this therapy not previously appreciated. It has recently been shown that TNF-α can stimulate the replication of AIEC in infected macrophages hence the anti-TNF-α mAb may also have directly impacted the growth and proliferation of AIEC in the *T. gondii* infection model. [61].

In summary, this study has explored the dynamics of the relationship between ileal inflammation and alterations in the intestinal microbiome termed dysbiosis. On the basis of our observations we propose a model where the composition and spatial distribution of the ileal microbiome is regulated by inflammation independent of the injurious trigger (Figure 4). Inflammation drives a progressive decrease in the microbial diversity or at the least a decrease in the evenness of the microbial population, a shift from Gram+ to Gram−, and the proliferation of mucosally invasive bacteria such as AIEC. The specific factors related to inflammation that induce dysbiosis remain to be elucidated. We speculate that inflammation related perturbations in the microenvironment such as increased availability of subtrates for growth of Gram −ve bacteria (e.g. iron and serum, dead or dying cells) and loss of niche and substrates for Gram +ve flora (e.g. mucus, goblet cells) underly this phenomenon. While the common end-point of inflammation is the inflammatory microbiome, genetic susceptibility can impact the threshold for dysbiosis in response to an external trigger. It seems plausible that genetic susceptibility may also influence the ability of an individual to resolve the self-perpetuating cycle of dysbiosis and inflammation generated by an acute insult.

## Supporting Information

Figure S1 **16S rDNA sequencing by genera. Moderate to severe ileitis in T8 and HDI induces a Gram negative shift dominated by >99%** ***Proteobacteria*** **and loss of microbial diversity, from 25 genera in controls, to 11 and 13 genera in T8 and HDI respectively.**
(TIFF)

Table S1 **Histopathological evaluation of ileal inflammation and mucosal morphology.**
(DOC)

Table S2 **16S rDNA pyrosequencing sequence classification (% of total sequence number, n) by genus for ileitis trigger experiments (5 mice per group): Control - uninfected/untreated mice; T4, T8 - 4 and 8 days after** ***T. gondii*** **infection; G7, G14 - 7 and 14 days after** ***G. muris*** **infection.** LDI, HDI: low dose (0.1 mg/mouse) and high dose (1 mg/mouse) indomethacin treatment.
(DOC)

Table S3 **16S rDNA pyrosequencing data for all mouse groups, showing Shannon-Weaver bacterial diversity index, observed operative taxonomical units (OTU), the predicted maximum number of OTUs, rarefaction, and species richness estimators (ACE and Chao 1) at strain (1% dissimilarity), species (3%), and genus (5%) level.**
(DOC)

Table S4 **16S rDNA pyrosequencing data (% of total sequence number, n) by genus for control, CCR2, NOD2 and anti-TNF-α mAb experiments (5 mice per group).**
(DOC)

Table S5 **Maximum Predicted Operational Taxonomic units at >10,000 sequences and calculated percent of maximum observed by rarefaction for each sample.**
(DOC)

Table S6 **PCR primers for** ***E. coli*** **genotypic characterization.**
(DOC)
