# Supplementary figures and images for: Inflammation Drives Dysbiosis and Bacterial Invasion in Murine Models of Ileal Crohn’s Disease
Source: PLoS One. 2012 Jul 25;7(7):e41594. doi: 10.1371/journal.pone.0041594 (PMC3404971; doi:10.1371/journal.pone.0041594)

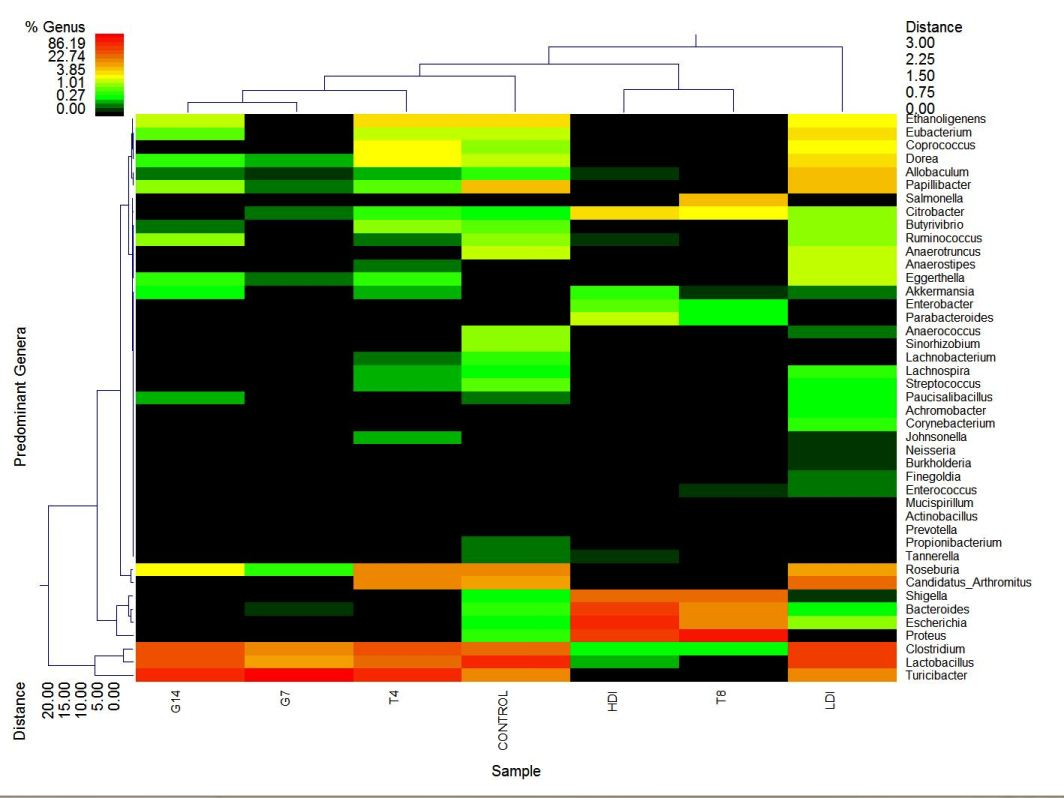

Supplement: Figure S1 — 16S rDNA sequencing by genera. Moderate to severe ileitis in T8 and HDI induces a Gram negative shift dominated by >99% Proteobacteria and loss of microbial diversity, from 25 genera in controls, to 11 and 13 genera in T8 and HDI respectively. (TIFF) [file pone.0041594.s001.tiff]
