# Supplementary material for: Inflammation Drives Dysbiosis and Bacterial Invasion in Murine Models of Ileal Crohn’s Disease
Source: PLoS One. 2012 Jul 25;7(7):e41594. doi: 10.1371/journal.pone.0041594 (PMC3404971; doi:10.1371/journal.pone.0041594)
Supplement: Table S1 — Histopathological evaluation of ileal inflammation and mucosal morphology. (DOC) [file pone.0041594.s002.doc]

Table S1: Histopathological evaluation of ileal inflammation and mucosal morphology

| ***Treatment*** | ***Group*** | ***ileitis score***  ***median (range)*** | ***difference within a treatment*** | ***Crypt: villus***  ***median (range)*** | ***difference within a treatment*** |
| --- | --- | --- | --- | --- | --- |
|  | **0** | **0 (0-0)** |  | **0.45 (.37-.65)** |  |
| **T. gondii** | **4** | **2 (0-2)** | **P=0.0028** | **0.49 (.48-.65)** | **P=0.007** |
|  | **8** | **4 (3-6) ***,##** |  | **1.16 (.74-1.56)***,##** |  |
|  | **0** | **0 (0-0)** |  | **0.35 (.31-.89)** |  |
| **Giardia** | **7** | **0 (0-0)** | **P=0.009** | **0.32 (.29-.38)** | **NSD** |
|  | **14** | **1 (1-1)***,###** |  | **0.30 (.26-.38)** |  |
|  | **0** | **0 (0-0)** |  | **0.45 (.37-.62)** |  |
| **Indomethacin** | **Low** | **2 (1-4)**** | **P=0.0036** | **0.46 (.37-.76)** | **NSD** |
|  | **High** | **5 (5-7)***** |  | **0.75 (.29-.94)** |  |
|  | **0** | **0 (0-0)** |  | **0.44 (.35-.52)** |  |
| **T. gondii CCR2 ^-/-^** | **4** | **0 (0-1)** | **P=0.0021** | **0.44 (.37-.53)** | **P=0.032** |
|  | **8** | **3 (2-3)***,#,^** |  | **0.91 (.83-1.18)***,###** |  |
|  | **0** | **0 (0-0)** |  | **0.64 (.456-.685)** |  |
| **T. gondii Nod2 ^-/-^** | **4** | **2 (1-4)*** | **P=0.0013** | **0.62 (.52-.84)** | **P=0.009** |
|  | **8** | **5 (5-7)***,#** |  | **1.44 (1.16-1.86)***,###** |  |
|  | **0** | **0 (0-0)** |  | **0.36 (.33-.43)** |  |
| **T. gondii anti-TNF** | **7** | **4 (2-4)**** | **P=0.0027** | **1.00 (.86-1.36)***** | **p=0.009** |
|  | **IgG7** | **4 (4-7)***** |  | **1.05 (.83-1.40)***** |  |

Differences within a treatment were evaluated by one way ANOVA (Kruskall–Wallis). Differences between groups within a treatment were only evaluated when Kruskall-Wallis P = <0.05 with significance is indicated vs. 0 as * = P <.05,** = P <0.01,*** = P <0.001, and vs. 4 (*T. gondii*) and 7 (Giardia) as # = P <.05, ## = P <0.01, ### = P<0.001.Difference vs. *T. gondii* 8 (Mann-Whitney): ^ = P <.05
