# Supplementary material for: Inflammation Drives Dysbiosis and Bacterial Invasion in Murine Models of Ileal Crohn’s Disease
Source: PLoS One. 2012 Jul 25;7(7):e41594. doi: 10.1371/journal.pone.0041594 (PMC3404971; doi:10.1371/journal.pone.0041594)
Supplement: Table S2 — 16S rDNA pyrosequencing sequence classification (% of total sequence number, n) by genus for ileitis trigger experiments (5 mice per group): Control - uninfected/untreated mice; T4, T8 - 4 and 8 days after T. gondii infection; G7, G14 - 7 and 14 days after G. muris infection. LDI, HDI: low dose (0.1 mg/mouse) and high dose (1 mg/mouse) indomethacin treatment. (DOC) [file pone.0041594.s003.doc]

Table S2: 16S rDNA pyrosequencing sequence classification (% of total sequence number, n) by genus for ileitis trigger experiments (5 mice per group): Control - uninfected/untreated mice; T4, T8 - 4 and 8 days after *T. gondii* infection; G7, G14 - 7 and 14 days after *G. muris* infection. LDI, HDI: low dose (0.1mg/mouse) and high dose (1mg/mouse) indomethacin treatment.

|  | **Mouse group** | | | | | | |
| --- | --- | --- | --- | --- | --- | --- | --- |
| **GENUS** | CONTROL  n=1898 | T4  n=1970 | T8  n=6101 | G7  n=6102 | G14  n=4099 | LD  n=2930 | HD  n=4903 |
| *Clostridium* | 13.4 | 17.1 | 0.2 | 7.4 | 22.6 | 30.2 | 0.1 |
| *Turicibacter* | 8.9 | 44.9 | 0.0 | 86.2 | 52.3 | 8.8 | 0.0 |
| *Lactobacillus* | 53.4 | 13.7 | 0.0 | 5.7 | 21.0 | 23.2 | 0.1 |
| *Roseburia* | 7.5 | 8.8 | 0.0 | 0.2 | 1.1 | 4.4 | 0.0 |
| *C. Arthromitus* | 4.7 | 7.7 | 0.0 | 0.0 | 0.0 | 13.1 | 0.0 |
| *Papillibacter* | 2.7 | 0.3 | 0.0 | 0.0 | 0.5 | 3.3 | 0.0 |
| *Anaerostipes* | 0.0 | 0.0 | 0.0 | 0.0 | 0.0 | 0.8 | 0.0 |
| *Coprococcus* | 0.5 | 1.2 | 0.0 | 0.0 | 0.0 | 1.4 | 0.0 |
| *Ethanoligenens* | 2.0 | 1.7 | 0.0 | 0.0 | 0.7 | 1.2 | 0.0 |
| *Allobaculum* | 0.2 | 0.1 | 0.0 | 0.0 | 0.0 | 3.4 | 0.0 |
| *Anaerotruncus* | 0.8 | 0.0 | 0.0 | 0.0 | 0.0 | 0.7 | 0.0 |
| *Eubacterium* | 0.8 | 0.9 | 0.0 | 0.0 | 0.3 | 1.7 | 0.0 |
| *Citrobacter* | 0.1 | 0.2 | 1.0 | 0.0 | 0.0 | 0.6 | 1.6 |
| *Escherichia* | 0.2 | 0.0 | 17.6 | 0.0 | 0.0 | 0.6 | 48.6 |
| *Proteus* | 0.2 | 0.0 | 71.1 | 0.0 | 0.0 | 0.0 | 24.6 |
| *Bacteroides* | 0.2 | 0.0 | 7.0 | 0.0 | 0.0 | 0.2 | 23.3 |
| Other | 4.1 | 3.2 | 3.1 | 0.2 | 1.1 | 6.2 | 1.5 |
