# Supplementary material for: Inflammation Drives Dysbiosis and Bacterial Invasion in Murine Models of Ileal Crohn’s Disease
Source: PLoS One. 2012 Jul 25;7(7):e41594. doi: 10.1371/journal.pone.0041594 (PMC3404971; doi:10.1371/journal.pone.0041594)
Supplement: Table S3 — 16S rDNA pyrosequencing data for all mouse groups, showing Shannon-Weaver bacterial diversity index, observed operative taxonomical units (OTU), the predicted maximum number of OTUs, rarefaction, and species richness estimators (ACE and Chao 1) at strain (1% dissimilarity), species (3%), and genus (5%) level. (DOC) [file pone.0041594.s004.doc]

Table S3: 16S rDNA pyrosequencing data for all mouse groups, showing Shannon-Weaver bacterial diversity index, observed operative taxonomical units (OTU), the predicted maximum number of OTUs, rarefaction, and species richness estimators (ACE and Chao 1) at strain (1% dissimilarity), species (3%), and genus (5%) level.
