# Supplementary material for: Inflammation Drives Dysbiosis and Bacterial Invasion in Murine Models of Ileal Crohn’s Disease
Source: PLoS One. 2012 Jul 25;7(7):e41594. doi: 10.1371/journal.pone.0041594 (PMC3404971; doi:10.1371/journal.pone.0041594)
Supplement: Table S4 — 16S rDNA pyrosequencing data (% of total sequence number, n) by genus for control, CCR2, NOD2 and anti-TNF-α mAb experiments (5 mice per group). (DOC) [file pone.0041594.s005.doc]

Table S4: 16S rDNA pyrosequencing data (% of total sequence number, n) by genus for control, CCR2, NOD2 and anti-TNF-α mAb experiments (5 mice per group).

|  | **Mouse group** | | | | | | | | | | | |
| --- | --- | --- | --- | --- | --- | --- | --- | --- | --- | --- | --- | --- |
| **GENUS** | Control  n=1898 | T4  n=1970 | T8  n=6101 | CCR2^-/-^0  n=3977 | CCR2^-/-^4  n=6102 | CCR2^-/-^8  n=1180 | NOD2^-/-^0  n=3579 | NOD2^-/-^4  n=3579 | NOD2^-/-^8  n-6102 | Anti-TNF-α-0  n=4949 | Anti-TNF-α-7 n=6101 | IgG-7 n=3652 |
| *Clostridium* | 4.3 | 0.5 | 0.0 | 6.9 | 0.3 | 3.7 | 1.1 | 0.0 | 0.0 | 5.3 | 1.5 | 0.1 |
| *Papillibacter* | 3.4 | 0.0 | 0.0 | 0.1 | 0.0 | 2.6 | 0.1 | 0.0 | 0.0 | 0.4 | 0.0 | 0.0 |
| *Shigella* | 0.0 | 0.0 | 4.2 | 0.0 | 0.0 | 6.3 | 0.1 | 0.0 | 4.1 | 0.0 | 0.0 | 0.0 |
| *Escherichia* | 0.0 | 0.0 | 14.4 | 0.0 | 0.0 | 8.7 | 0.2 | 0.0 | 93.3 | 0.0 | 0.0 | 0.0 |
| *Dorea* | 2.3 | 0.2 | 0.0 | 0.6 | 0.1 | 0.3 | 0.7 | 0.0 | 0.0 | 0.7 | 0.0 | 0.0 |
| *Streptococcus* | 0.0 | 1.1 | 0.0 | 0.0 | 0.0 | 0.0 | 0.0 | 1.3 | 0.0 | 0.0 | 0.0 | 0.0 |
| *Tannerella* | 0.1 | 0.0 | 0.0 | 7.8 | 5.1 | 0.0 | 46.0 | 7.0 | 0.0 | 21.3 | 1.1 | 0.0 |
| *Turicibacter* | 23.0 | 71.2 | 0.0 | 79.4 | 93.3 | 2.5 | 42.2 | 60.1 | 0.0 | 52.0 | 0.0 | 0.0 |
| *Citrobacter* | 0.2 | 0.0 | 0.0 | 0.1 | 0.0 | 0.4 | 0.2 | 0.0 | 2.5 | 0.1 | 0.0 | 0.0 |
| *Proteus* | 0.0 | 0.1 | 75.3 | 0.0 | 0.0 | 41.5 | 0.0 | 0.0 | 0.0 | 5.8 | 76.1 | 99.7 |
| *Anaerotruncus* | 1.4 | 0.0 | 0.0 | 0.2 | 0.0 | 0.2 | 0.0 | 0.0 | 0.0 | 0.0 | 0.0 | 0.0 |
| *Roseburia* | 3.8 | 0.1 | 0.0 | 2.3 | 0.1 | 1.8 | 1.2 | 0.0 | 0.0 | 1.1 | 0.0 | 0.0 |
| *Parabacteroides* | 0.3 | 0.0 | 0.0 | 0.0 | 0.0 | 0.4 | 0.0 | 0.0 | 0.0 | 0.5 | 19.8 | 0.1 |
| *Lactobacillus* | 20.9 | 18.4 | 0.0 | 0.7 | 0.3 | 0.1 | 5.4 | 28.4 | 0.0 | 10.1 | 0.1 | 0.0 |
| *C. Arthromitus* | 32.5 | 4.3 | 0.0 | 0.0 | 0.0 | 0.0 | 0.0 | 0.0 | 0.0 | 0.0 | 0.0 | 0.0 |
| *Bacteroides* | 1.0 | 0.0 | 2.6 | 0.0 | 0.0 | 26.3 | 0.0 | 0.0 | 0.0 | 0.0 | 0.1 | 0.0 |
| Other | 6.8 | 4.1 | 3.5 | 1.7 | 0.6 | 5.1 | 2.9 | 3.3 | 0.2 | 2.7 | 1.3 | 0.1 |
