# Supplementary material for: Inflammation Drives Dysbiosis and Bacterial Invasion in Murine Models of Ileal Crohn’s Disease
Source: PLoS One. 2012 Jul 25;7(7):e41594. doi: 10.1371/journal.pone.0041594 (PMC3404971; doi:10.1371/journal.pone.0041594)
Supplement: Table S5 — Maximum Predicted Operational Taxonomic units at >10,000 sequences and calculated percent of maximum observed by rarefaction for each sample. (DOC) [file pone.0041594.s006.doc]

Table S5: Maximum Predicted Operational Taxonomic units at > 10,000 sequences and calculated percent of maximum observed by rarefaction for each sample

| Sample | Max Predicted 10,000 | Max Predicted 15,000 | Max Predicted 20,000 | Percent coverage of observed |
| --- | --- | --- | --- | --- |
| Control | 71 | 71 | 71 | 97 |
| T4 | 76 | 76 | 76 | 93 |
| T8 | 29 | 29 | 29 | 100 |
| G7 | 55.5 | 56 | 56 | 80 |
| G14 | 75 | 75 | 75 | 92 |
| LDI | 131 | 132 | 132 | 86 |
| HDI | 32 | 32 | 32 | 100 |
| CCR2-0 | 80 | 80 | 80 | 90 |
| CCR2-4 | 47 | 47 | 47 | 79 |
| CCR2-8 | 72* | 72* | 72* | 89 |
| NOD2-0 | 79 | 79 | 80 | 88 |
| NOD2-4 | 52 | 52 | 52 | 83 |
| NOD2-8 | 8 | 8 | 8 | 100 |
| TNF-0 | 113 | 113 | 113 | 90 |
| TNF-7 | 27 | 27 | 27 | 96 |
| IgG-7 | 5 | 5 | 5 | 100 |
