# Supplementary material for: Inflammation Drives Dysbiosis and Bacterial Invasion in Murine Models of Ileal Crohn’s Disease
Source: PLoS One. 2012 Jul 25;7(7):e41594. doi: 10.1371/journal.pone.0041594 (PMC3404971; doi:10.1371/journal.pone.0041594)
Supplement: Table S6 — PCR primers for E. coli genotypic characterization. (DOC) [file pone.0041594.s007.doc]

Table S6: PCR primers for *E. coli* genotypic characterization

| Gene | Primer sequence (5'-3') | Amplicon size (bp) | Reference |
| --- | --- | --- | --- |
| ratA | ratA F: aac gtc ttt ctc atc cga ggc agt  ratA R: tgt cga taa aga agc gcc cga tgt | 409 | 20 |
| PmtI | PmtI F: ttt cac ctt ccg cga cca cat cta  PmtI R: tcc tcc aac ttc agc gga atc aca | 301 | 20 |
| colV | colV F: aag ttc tca atc gtc tgg cct gga  colV R: ggc ggc atc cat cat tgc ttt aca | 302 | 20 |
| Hcp | Hcp F: gca ggt tgc tca acg ctt gat tct  Hcp R: tac ccg ctt tac tgg ttc ggt aga | 235 | 20 |
| lpfA141 | lpfA141 F: gca cca ttt gta taa tct gcg ccc  lpfA141 R: tga gag atg atc gtt ccg tca gga | 627 | This study |
| lpfA154 | lpfA154-F: gcc gtc tct ttg gtg tac tat tcc  lpfA154-R: cca cca atg aca acg cct gca taa | 846 | This study |
| kpsMII | kpsmII-F: gcgcatttgctgatactgttg  kpsmII-R: catccagacgataagcatgagca | 272 | 34 |
| fyuA | fyuA-F: ggcggcgtgcgcttctcgca  fyuA-R: cgcagtaggcacgatgttgta | 209 | 32 |
| Iss | issF: gtg gcg aaa act agt aaa aca gc  issR : cgc ctc ggg gtg gat aa | 762 | 33 |
| malX | malX-F: ggacatcctgttacagcgcgca  malX-R: tcgccaccaatcacagccgaac | 930 | 34 |
| Gsp | Gsp F: ggc gaa ctg gaa acc atg ctt gag  Gsp R:att gtt cag ttg cag cat cgg ctc | 171 | This study |
| traC | traC F: gtt tgt gca gca gaa gcg taa aga g  traC R: ttc tca tgt tct tcc agc cag gct tc | 120 | This study |
| afa | afaB: gct ggg cag caa act gat aac tct c  afaC:cat caa gct gtt tgt tcg tcc gcc g | 750 | 33 |
| focG | focG F: cag cac agg cag tgg atac ga  focG R: gaa tgt cgc ctg ccc att gct | 360 | 34 |
| ibeA | ibeA F: tgaacgtttcggttgttttg  ibeA R: tgttcaaatcctggctggaa | 814 | 33 |
| papC | papC F: gac ggc tgt act gca ggg tgt ggc g  papC R: ata tcc ttt ctg cag gga tgc aat a | 328 | 33 |
| sfaDE | sfaD: ctc cg gaga act ggg tgc atc tta c  sfaE: cgg agg agt aat tac aaa cct ggc a | 410 | 33 |
